# A neuro swarm procedure to solve the novel second order perturbed delay Lane-Emden model arising in astrophysics

**DOI:** 10.1038/s41598-022-26566-4

**Published:** 2022-12-30

**Authors:** Zulqurnain Sabir, Salem Ben Said, Qasem Al-Mdallal, Mohamed R. Ali

**Affiliations:** 1 Department of Mathematical Science, College of Science, United Arab Emirates University, P. O. Box 15551, Al Ain, United Arab Emirates; 2 Faculty of Engineering and Technology, Future University in Egypt, New Cairo, 11835 Egypt; 3 Basic Engineering Science Department, Benha Faculty of Engineering, Benha University, Benha, Egypt

**Keywords:** Environmental social sciences, Astronomy and planetary science, Mathematics and computing

## Abstract

The current work provides a mathematical second order perturbed singular delay differential model (SO-PSDDM) by using the standard form of the Lane-Emden model. The inclusive structures based on the delay terms, singular-point and perturbation factor and shape forms of the SO-PSDDM are provided. The novel form of the SO-PSDDM is numerically solved by using the procedures of artificial neural networks (ANNs) along with the optimization measures based on the swarming procedures (PSO) and interior-point algorithm (IPA). An error function is optimized through the swarming PSO procedure along with the IPA to solve the SO-PSDDM. The precision, substantiation and validation are observed for three problems of the SO-PSDDM. The exactness of the novel SO-PSDDM is observed by comparing the obtained and exact solutions. The reliability, stability and convergence of the proposed stochastic algorithms are observed for 30 independent trials to solve the novel SO-PSDDM.

## Introduction

The solutions of the singular models always a big challenge for the scientists due to the reason of the singular point that arise at origin. These systems become stiffer and complicated by using the perturbed terms with the boundary layer performance. The singular types present the speedy disparities to conduct the thin boundary layers. There are some typical techniques that present the perturbation form of the singularity, but these schemes fail to achieve the appropriate solutions due to the small perturbation factors. Consequently, it is the need of the time to design some reliable numerical schemes for such models^1–7^. A computing scheme using the finite difference and the exponential fitting is explored to achieve the performances of the singular perturbed form of the models^8–10^. Some other schemes presented to solve the convection–diffusion second order perturbed singular delay differential model (SO-PSDDM)^11^. The mesh approach using several simulations has been used to solve the reaction–diffusion models^9,12^ and the diffusion reaction semi-linear based models have been solved in^13^.

The use of delay factor is considered very important due for the researchers due to its enormous applications in the biological sciences, ships controlling, number theory, light absorption in the stellar matter, medical field, chemistry, electronics, infectious diseases, physical systems, quantum mechanics, economics, engineering, electrodynamics and control systems^14^. The researchers are always interested to solve these models due to these mentioned applications. Perko^15^ designed the linear and nonlinear types of the differential models using the dynamical constructions. Lasalle^16^ and Kuang^17^ investigated the solution methods together with the delay form of the models. Forde^18^ presented the delay biological systems and Beretta et al.^19^ introduced the geometric dependability using the terms based on delay dependent.

The differential singular models have a variety of applications in quantum mechanics, gas cloud and astrophysics^20–23^. There are many scientists that are interested to solve the singular differential models, as these models are always challenging to handle for the researchers. Most of the traditional schemes do not work to solve such singular models, so different approximations have been applied to solve these singular models. Therefore, artificial neural networks (ANNs) optimized by the global and local search schemes are a better choice to solve the singular models, because it gives solutions at exact singularity without any approximation. One important singular model is known as Lane-Emden model that is famous due to the historical aspects. The mathematical form of this historical model is written as^24,25^:

1 $$\left\{ \begin{array}{ll} \frac{{d^{2} z}}{{du^{2} }} + \frac{\omega }{u}\frac{dz}{{du}} + r(z) = v(u), \quad & \omega \ge 1 \hfill \\ z(0) = a, &\quad \frac{dz(0)}{{du}} = 0, \hfill \\ \end{array} \right.$$

where $\omega$ represents the shape factor, the singular point is at $u = 0$, *r*(*z*) and *v*(*u*) are the functions of *z* and *u*. The purpose of these investigations is to present the numerical solutions of the novel SO-PSDDM by using the artificial neural networks (ANNs) along with the optimization measures of the swarming procedures (PSO) and interior-point algorithm (IPA). The novel features for solving the new designed SO-PSDDM are provided as:

- A novel mathematical singular kind of system is constructed with the perturbed, standard Lane-Emden and delay terms.
- Soft computing approaches using the process of ANNs process along with the optimal structures of the swarming scheme and interior-point algorithm have been presented for solving the novel SO-PSDDM.
- The computing competence of the ANNs procedure using the optimal structures of the swarming scheme and IPA is provided for three different problems of the novel SO-PSDDM.
- The obtained and exact results have been compared to prove the correctness of the computational stochastic approach.
- The absolute error (AE) measures are provided in good actions, which validates the precision of the swarming computational scheme.
- The dependability and reliability of proposed ANNs procedure using the optimization structures of the swarming scheme and IPA are observed for solving the novel SO-PSDDM by using the statistical presentations of the semi-interquartile range (SIR), Theil inequality coefficient (TIC) and mean square error (MSE).
- Beside the detailed structure of the mathematical model and designing of the stochastic scheme, constancy, robustness, smooth actions, inclusive pertinency and ease of understanding are other noteworthy perks.

A novel SO-PSDDM is proposed in this study, which becomes stiffer and more complex by using the delay and perturbed factors. Therefore, a stochastic ANNs computing framework using the optimal structures of the particle swarm optimization (PSO) and IPA is presented to solve the novel SO-PSDDM. Recently, the computational stochastic solvers have notable submissions to solve the fractional and integer kinds of systems. Some important applications of the stochastic solvers are coronavirus model^26^, stomach model^27^, dengue fever model^28^, the mosquito form of the spreading ecosystem^29^, food supply chain nonlinear systems^30,31^, HIV anticipation model^32^, HIV infection system^33^ and medical smoking system^34,35^.

The other parts of this study are presented as: Section "A novel design of SO-PSDDM" shows the design of the novel SO-PSDDM. Section "Methodology:" indicates the stochastic methodology. Section "Results and Simulations" shows the results of the novel SO-PSDDM, and the concluding remarks are listed in the last Section.

## A novel design of SO-PSDDM

This section shows the novel design of the SO-PSDDM by using the terminology of the Lane-Emden, delay model and perturbed terms. Recently, many systems that have been designed with the terminology of the Lane-Emden model. Few of them are the singular 2nd and 3rd pantograph model, 4th, 5th order and 6th kinds of functional singular systems, prediction, delay and pantograph form of the singular models^36–44^. Based on these applications, the authors are inspired to construct a novel SO-PSDDM. The perturbed delay form of the singular differential model is mathematically given as:

2 $$\varepsilon u^{ - \omega } \frac{{d^{\eta } }}{{du^{\eta } }}\left( {u^{\omega } \frac{{d^{\chi } }}{{du^{\chi } }}} \right)z\left( {u - \varphi } \right) + r(z) = v(u),$$

where $\varepsilon$ is the perturbed terms and $\omega$ is taken as positive. For the SO-PSDDM, $\chi$ and $\eta$ values are provided as:

3 $$\chi = 1,\,\,\,\eta = 1.$$

By using the above values, the Eq. (2) takes the form as:

4 $$\varepsilon u^{ - \omega } \frac{d}{du}\left( {u^{\omega } \frac{d}{du}} \right)z\left( {u - \varphi } \right) + r(z) = v(u).$$

The simplified form of the $\frac{d}{du}\left( {u^{\omega } \frac{d}{du}} \right)z\left( {u - \varphi } \right)$ is given as:

5 $$\frac{d}{du}\left( {u^{\omega } \frac{d}{du}} \right)z\left( {u - \varphi } \right) = u^{\omega } \frac{{d^{2} }}{{du^{2} }}z\left( {u - \varphi } \right) + \omega u^{\omega - 1} \frac{d}{du}z\left( {u - \varphi } \right).$$

The achieved SO-PSDDM is provided as:

6 $$\left\{ \begin{gathered} \varepsilon \frac{{d^{2} }}{{du^{2} }}z\left( {u - \varphi } \right) + \varepsilon \frac{\omega }{u}\frac{d}{du}z\left( {u - \varphi } \right) + r(z) = v(u), \hfill \\ u(0) = 1,\,\,\,\frac{d}{du}(0) = 0.\,\,\, \hfill \\ \end{gathered} \right.$$

**Figure 1:**
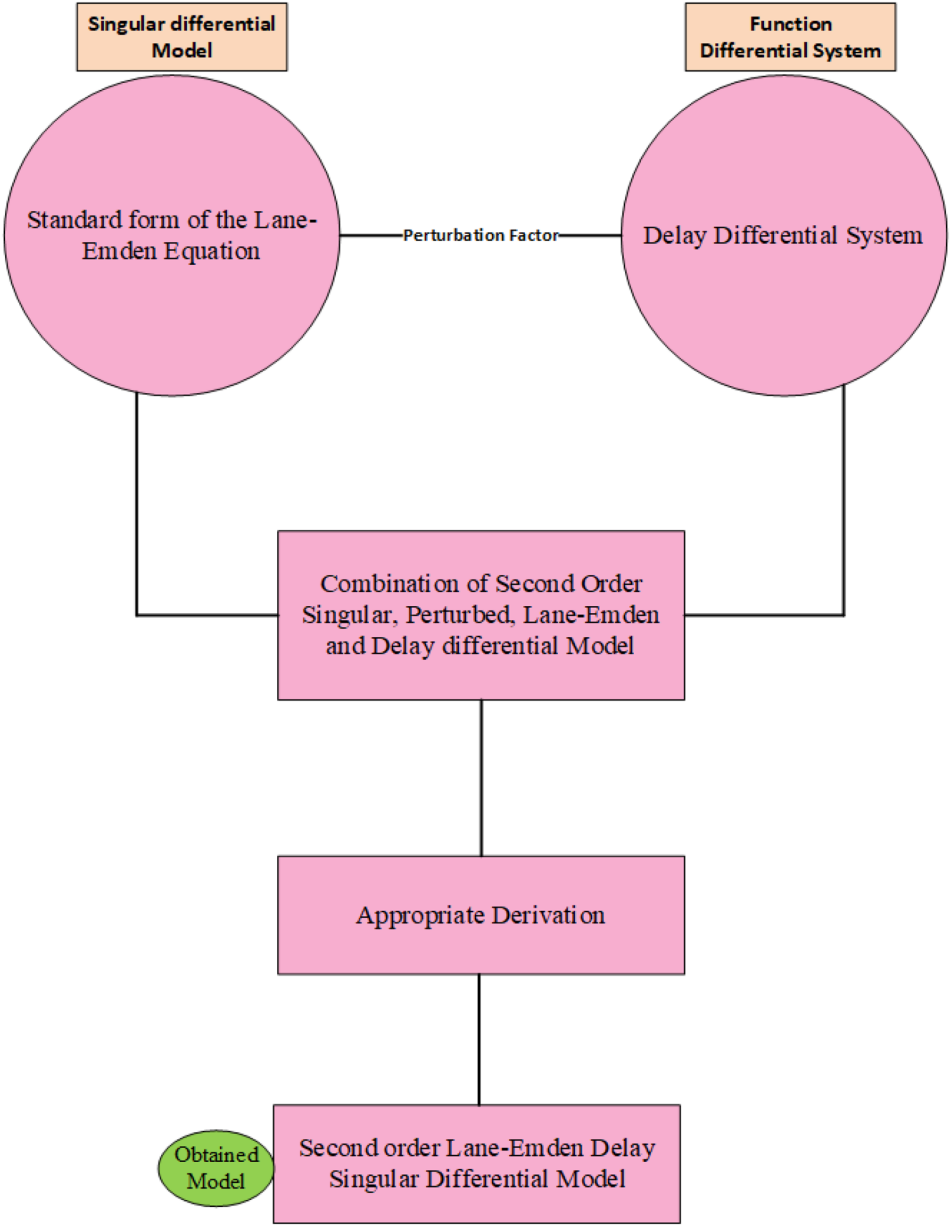
Description of Flow-chart for solving the novel SO-PSDDM.

Equation (6) represents the novel SO-PSDDM, $\varepsilon$ is the perturbed factor and $\varphi$ shows the delay term. The perturbed and delay terms appear twice, while single singularity and shape factor is noticed in the Eq. (6). The detailed descriptions of the flow-chart based on the design of novel SO-PSDDM is presented in Fig. 1.

## Methodology

**Figure 2:**
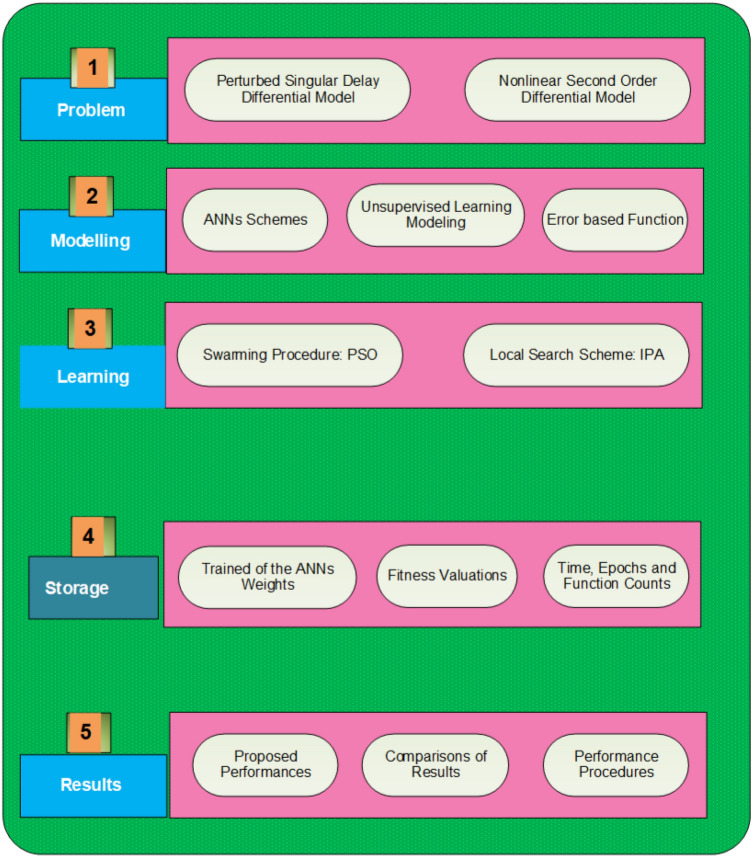
Designed stochastic procedure for solving the novel design of the SO-PSDDM.

The current section represents the ANNs process using the optimal structure of the swarming scheme and interior-point algorithm for the SO-PSDDM. The computational structure for the novel design of the SO-PSDDM is shown in Fig. 2.

### Formulations of the ANNs procedure

For the solutions of the novel SO-PSDDM, the proposed results are indicated as $\hat{z}(u)$, given as:

7 $$\hat{z}(u) = \sum\limits_{k = 1}^{p} {m_{k} R(w_{k} u + y_{k} )} ,$$

$$\frac{{d^{(n)} \hat{z}}}{{du^{(n)} }} = \sum\limits_{k = 1}^{p} {m_{k} \frac{{d^{(n)} }}{{du^{(n)} }}R(w_{k} u + y_{k} )} ,$$

here $[m_{k} ,w_{k} ,y_{k} ]$ represent the *k*th components of $[{\varvec{m}},{\varvec{w}},{\varvec{y}}]$ and *p* is the neuron. An activation log-sigmoid function $R(u) = (1 + e^{ - u} )^{ - 1}$ is used in the Eq. (7) as:

8 $$\begin{gathered} \hat{z}(u) = \sum\limits_{k = 1}^{p} {m_{k} \left( {1 + e^{{ - (w_{k} u + y_{k} )}} } \right)^{ - 1} } , \hfill \\ \frac{{d\hat{z}}}{du} = \sum\limits_{k = 1}^{p} {m_{k} w_{k} \frac{{e^{{ - (w_{k} u + y_{k} )}} }}{{\left( {1 + e^{{ - (w_{k} u + y_{k} )}} } \right)^{2} }}} , \hfill \\ \vdots \hfill \\ \frac{{d^{(n)} \hat{z}}}{{du^{(n)} }} = \sum\limits_{k = 1}^{p} {m_{k} w_{k} \left( {\frac{{e^{{ - (w_{k} u + y_{k} )}} }}{{\left( {1 + e^{{ - (w_{k} u + y_{k} )}} } \right)^{n + 1} }} - \frac{{e^{{ - \left( {n + 1} \right)(w_{k} u + y_{k} )}} }}{{\left( {1 + e^{{ - (w_{k} u + y_{k} )}} } \right)^{n} }} \cdots } \right)} . \hfill \\ \end{gathered}$$

An error function ($\varepsilon_{f}$) is provided as:

9 $$\varepsilon_{f} = \varepsilon_{f-1} + \varepsilon_{f-2} ,$$

where, $\varepsilon_{f-1}$ and $\varepsilon_{f-2}$ shows the error functions based on the novel SO-PSDDM presented in the Eq. (6). The values of the $\varepsilon_{f}$ are presented as:

10 $$\varepsilon_{f} = \frac{1}{N}\sum\limits_{k = 1}^{N} {\left( {\varepsilon \frac{{d^{2} }}{{du_{k}^{2} }}\hat{z}\left( {u_{k} - \varphi } \right) + \varepsilon \frac{\omega }{{u_{k} }}\frac{d}{{du_{k} }}\hat{z}\left( {u_{k} - \varphi } \right) + r(\hat{z}) - v(u_{k} )} \right)^{2} } \, + \frac{1}{2}\left( {\left( {\hat{z}_{0} - 1} \right)^{2} + \left( {\frac{{d\hat{z}_{0} }}{{du_{k} }}} \right)^{2} } \right),$$

where $$Nh = 1,\,\,\hat{z}(u_{k} - \varphi ) = z\left( {u - \varphi } \right),\,\,r(\hat{z}) = r\left( z \right)$$ and $$v\left( {u_{k} } \right) = v\left( u \right)$$.

### Performance indices

The statistical SIR, TIC and MSE operators for the novel design of the SO-PSDDM using the stochastic scheme, given as:

11 $${\text{SIR}} = - \frac{1}{2}\left( {1^{{{\text{st}}}} {\text{Quartile}} - {3}^{{{\text{rd}}}} \,{\text{Quartile}}} \right),$$

12 $${\text{TIC = }}\frac{{\sqrt {\frac{1}{n}\sum\limits_{j = 1}^{k} {\left( {z_{j} - \hat{z}_{j} } \right)^{2} } } }}{{\left( {\sqrt {\frac{1}{n}\sum\limits_{j = 1}^{k} {z_{j}^{2} } } + \sqrt {\frac{1}{n}\sum\limits_{j = 1}^{k} {\hat{z}_{j}^{2} } } } \right)}},$$

13 $${\text{MSE = }}\sum\limits_{j = 1}^{k} {\left( {z_{j} - \hat{z}_{j} } \right)^{2} } ,$$

where $\hat{z}$ and *z* are the proposed and true solutions.

### Network optimization

The optimization parameters for solving the novel design of the SO-PSDDM using the ANNs procedures based on the optimization structures of the swarming scheme and IPA.

PSO is a computational Neuro swarming global search scheme that is used as an optimization algorithm. PSO provides the solutions of the various complex models to adjust the accurate population through the optimal training procedure. PSO is applied as a replacement of the global genetic algorithm. PSO is introduced by Kennedy and Eberhart in the end of the nineteenth century^45^. The PSO execution process is simple due to its short memory requirements^46^. Recently, PSO is applied in various applications, e.g., multimodal multi-objective optimization^47^, mixed-variable optimization problems^48^, solar energy systems^49^, engineering problems^50^, plant diseases diagnosis^51^, architectures for image classification^52^, identifying the single, double, and three diode photovoltaic models’ parameters^53^, particle filter noise reduction in mechanical fault diagnosis^54^ and green coal production problem^55^. These extraordinary applications impressed the authors to present the solutions of the novel design of the SO-PSDDM using the ANNs procedures based on the optimization structures of the swarming scheme.

As the process of global search PSO is slow, so the sluggishness and laziness of this scheme are improved by using the local search procedure to find the best convergence. Therefore, IPA is applied as a local refinement to find the rapid outcomes. The optimal PSO presentations are applied as a primary input in the IPA. Recently, IPA is used in the phase-field approach to brittle and ductile fracture^56^, power system observability^57^, multipliers for linear positive semi-definite programming^58^, simulation and optimization of dynamic flux balance analysis models^59^, fourth order singular systems^60^, multistage nonlinear nonconvex programs^61^ and monotone weighted linear complementarity problems^62^.

## Results and Simulations

This section presents the solutions of the SO-PSDDM by using the ANNs procedures based on the optimization structures of the swarming scheme and IPA. Thirty numbers of executions have been performed to validate the trustworthiness of the computational procedure for solving the SO-PSDDM.

### Problem 1:

Suppose the SO-PSDDM in Eq. (6) with $\varphi = \frac{1}{3}$,$\varepsilon = \frac{1}{{2^{3} }}$, $\omega = 2$ and $r(z) = z^{2}$ is given as:

14 $$\left\{ \begin{gathered} \frac{1}{8}\frac{{d^{2} }}{{du^{2} }}z\left( {u - \frac{1}{3}} \right) + \frac{1}{4u}\frac{d}{du}z\left( {u - \frac{1}{3}} \right) + z^{2} = v(u), \hfill \\ u(0) = 1,\,\,\,\frac{d}{du}(0) = 0,\, \hfill \\ \end{gathered} \right.$$

where $v(u) = u^{4} + 2u^{2} - \frac{1}{6u} + \frac{7}{4}$. The exact solution is $z(u) = 1 + u^{2}$ and an error function is given as:

15 $$\varepsilon_{f} = \frac{1}{N}\sum\limits_{k = 1}^{N} {\left( {\frac{1}{8}\frac{{d^{2} }}{{du_{k}^{2} }}\hat{z}\left( {u_{k} - \frac{1}{3}} \right) + \frac{1}{{4u_{k} }}\frac{d}{{du_{k} }}\hat{z}\left( {u_{k} - \frac{1}{3}} \right) + \hat{z}^{2} - v(u_{k} )} \right)^{2} } \, + \frac{1}{2}\left( {\left( {\hat{z}_{0} - 1} \right)^{2} + \left( {\frac{{d\hat{z}_{0} }}{{du_{k} }}} \right)^{2} } \right).$$

### Problem 2:

Suppose a novel design of the SO-PSDDM in Eq. (6) with $\varphi = \frac{1}{3}$,$\varepsilon = \frac{1}{{2^{5} }}$, $\omega = 2$ and $r(z) = z^{2}$ in shown as:

16 $$\left\{ \begin{gathered} \frac{1}{32}\frac{{d^{2} }}{{du^{2} }}z\left( {u - \frac{1}{3}} \right) + \frac{1}{16u}\frac{d}{du}z\left( {u - \frac{1}{3}} \right) + z^{2} = v(u), \hfill \\ u(0) = 1,\,\,\,\frac{d}{du}(0) = 0,\, \hfill \\ \end{gathered} \right.$$

where $v(u) = u^{6} + 2u^{3} + \frac{3}{8}u + \frac{1}{48u} + \frac{13}{{16}}$. The true results are $z(u) = 1 + u^{3} .$ The error function is provided as:

17 $$\varepsilon_{f} = \frac{1}{N}\sum\limits_{k = 1}^{N} {\left( {\frac{1}{32}\frac{{d^{2} }}{{du_{k}^{2} }}\hat{z}\left( {u_{k} - \frac{1}{3}} \right) + \frac{1}{{16u_{k} }}\frac{d}{{du_{k} }}\hat{z}\left( {u_{k} - \frac{1}{3}} \right) + \hat{z}^{2} - v(u_{k} )} \right)^{2} } \, + \frac{1}{2}\left( {\left( {\hat{z}_{0} - 1} \right)^{2} + \left( {\frac{{d\hat{z}_{0} }}{{du_{k} }}} \right)^{2} } \right).$$

### Problem 3

Suppose a novel design of the SO-PSDDM in Eq. (6) with $\varphi = \frac{1}{3}$,$\varepsilon = \frac{1}{{2^{7} }}$, $\omega = 2$ and $r(z) = z^{2}$ is written as:

18 $$\left\{ \begin{gathered} \frac{1}{128}\frac{{d^{2} }}{{du^{2} }}z\left( {u - \frac{1}{3}} \right) + \frac{1}{64u}\frac{d}{du}z\left( {u - \frac{1}{3}} \right) + z^{2} = v(u), \hfill \\ u(0) = 1,\,\,\,\frac{d}{du}(0) = 0, \hfill \\ \end{gathered} \right.$$

where $$v(u) = u^{8} + 2u^{4} + \frac{5}{32}u^{2} - \frac{1}{8}u + \frac{99}{{96}} - \frac{1}{432u}$$.

The true form of the above equation is $z(u) = 1 + u^{4} .$ A merit function is shown as:

19 $$\varepsilon_{f} = \frac{1}{N}\sum\limits_{k = 1}^{N} {\left( {\frac{1}{128}\frac{{d^{2} }}{{du_{k}^{2} }}\hat{z}\left( {u_{k} - \frac{1}{3}} \right) + \frac{1}{{64u_{k} }}\frac{d}{{du_{k} }}\hat{z}\left( {u_{k} - \frac{1}{3}} \right) + \hat{z}^{2} - v(u_{k} )} \right)^{2} } \, + \frac{1}{2}\left( {\left( {\hat{z}_{0} - 1} \right)^{2} + \left( {\frac{{d\hat{z}_{0} }}{{du_{k} }}} \right)^{2} } \right).$$

The optimization is performed for each example of the novel design of the SO-PSDDM using the ANNs and the optimization structures of the swarming scheme and IPA. Thirty executions have been implemented to validate the constancy of the designed scheme for the novel s SO-PSDDM. The proposed solutions based on the stochastic schemes are accomplished to achieve the unidentified weight vectors are presented as:

20 $$\begin{gathered} \hat{z}_{P - 1} (u) = \frac{ - 1.3378}{{1 + e^{ - ( - 0.6390u + 1.3003)} }} + \frac{1.6912}{{1 + e^{ - (1.0511u - 1.7052)} }} + \frac{0.8748}{{1 + e^{ - ( - 0.8417u + 1.0828)} }} + \frac{1.0631}{{1 + e^{ - ( - 1.0096u - 0.2519)} }} \hfill \\ \,\,\,\,\,\,\,\,\,\,\,\,\,\,\,\,\, + \frac{2.6159}{{1 + e^{ - ( - 2.8684u - 4.4948)} }} + \frac{0.5429}{{1 + e^{ - (0.1891u + 3.0291)} }} + \frac{1.2144}{{1 + e^{ - ( 2.0158u - 1.5849)} }} + \frac{1.9727}{{1 + e^{ - ( - 2.0326u - 1.7495)} }} \hfill \\ \,\,\,\,\,\,\,\,\,\,\,\,\,\,\,\,\, - \frac{0.6228}{{1 + e^{ - ( - 0.8033u + 0.9368)} }} + \frac{3.9092}{{1 + e^{ - ( 2.2451u - 2.9373)} }}, \hfill \\ \end{gathered}$$

21 $$\begin{gathered} \hat{z}_{P - 2} (u) = \frac{ - 1.5610}{{1 + e^{ - ( - 2.2216u + 0.1342)} }} + \frac{0.3299}{{1 + e^{ - (1.5883u + 0.7514)} }} - \frac{1.6561}{{1 + e^{ - ( - .1.2447u + 3.4474)} }} + \frac{1.1063}{{1 + e^{ - ( - 0.1879u - 1.1985)} }} \hfill \\ \,\,\,\,\,\,\,\,\,\,\,\,\,\,\,\,\, + \frac{2.9749}{{1 + e^{ - ( 2.8000u + 2.9488)} }} + \frac{0.2102}{{1 + e^{ - (1.3728u - 9.2328)} }} + \frac{4.1024}{{1 + e^{ - ( 2.6258u - 5.2880)} }} - \frac{1.6973}{{1 + e^{ - ( 1.1943u - 3.8500)} }} \hfill \\ \,\,\,\,\,\,\,\,\,\,\,\,\,\,\,\,\, + \frac{7.7997}{{1 + e^{ - ( 2.4388u - 3.9663)} }} + \frac{0.0052}{{1 + e^{ - ( - 12.6701u - 3.6229)} }}, \hfill \\ \end{gathered}$$

22 $$\begin{gathered} \hat{z}_{P - 3} (u) = \frac{ - 1.5320}{{1 + e^{ - ( - 0.4564u - 0.8733)} }} + \frac{4.9872}{{1 + e^{ - ( - 2.0420u - 3.2741)} }} + \frac{6.7268}{{1 + e^{ - (4.3029u - 6.2221)} }} + \frac{1.5267}{{1 + e^{ - ( 2.3256u - 0.0639)} }} \hfill \\ \,\,\,\,\,\,\,\,\,\,\,\,\,\,\,\,\, + \frac{2.2852}{{1 + e^{ - ( 1.5985u + 18.831)} }} - \frac{0.5827}{{1 + e^{ - (0.7475u + 2.5703)} }} - \frac{0.0661}{{1 + e^{ - ( - 1.2763u + 0.2649)} }} + \frac{2.8056}{{1 + e^{ - ( 1.6485u - 2.3113)} }} \hfill \\ \,\,\,\,\,\,\,\,\,\,\,\,\,\,\,\,\, - \frac{0.9169}{{1 + e^{ - ( 1.4073u + 19.8177)} }} - \frac{1.9480}{{1 + e^{ - ( 2.8740u - 0.9959)} }}, \hfill \\ \end{gathered}$$

**Figure 3:**
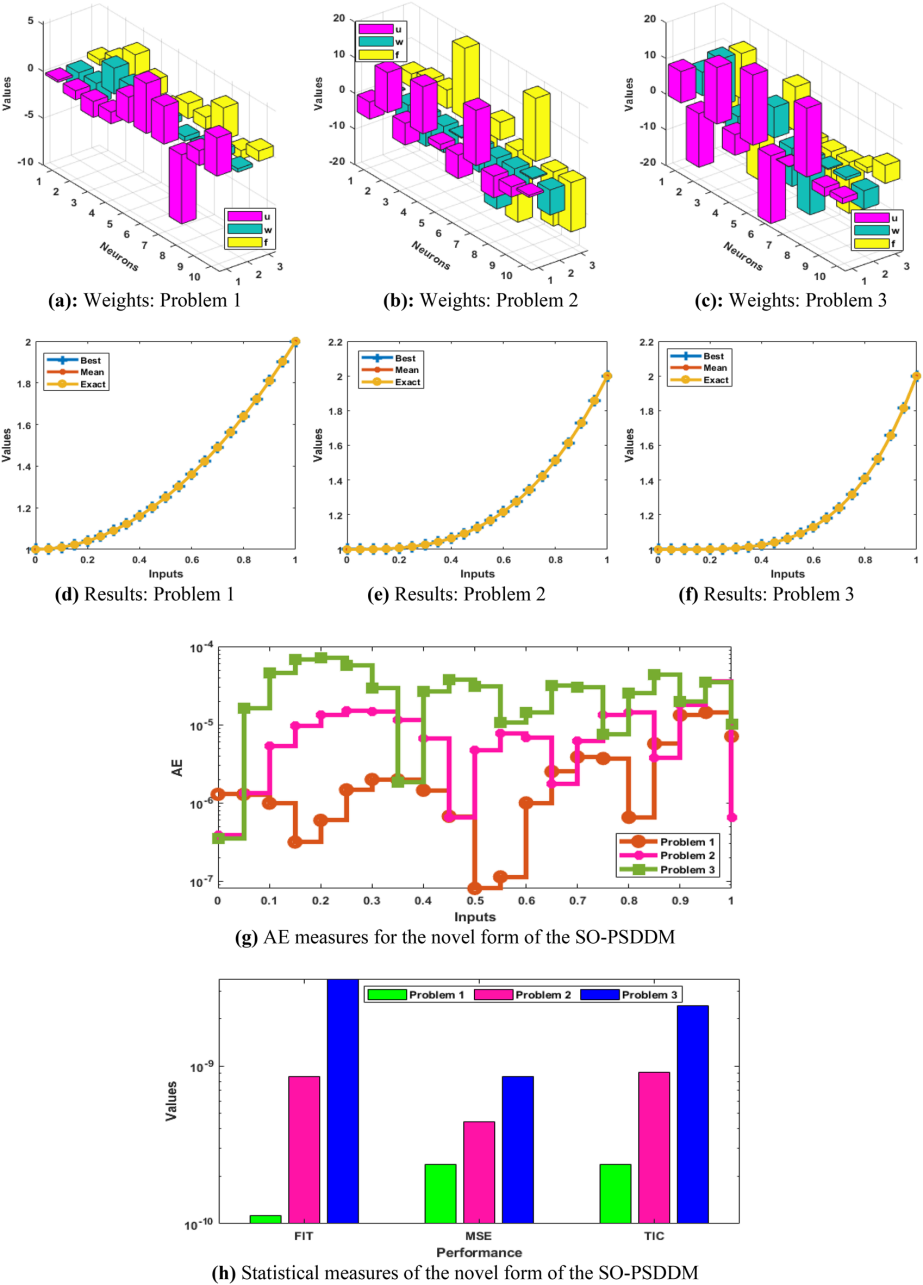
Graphic representations of optimal weight vectors, AE and statistical performances for solving the SO-PSDDM.

The achieved results through the stochastic scheme are presented in the set of Eq. (20–22). These numerical values have been plotted in Fig. 3 based on the best weight vectors to solve the novel SO-PSDDM. The results comparison performances based on the worst, best and mean outcomes are drawn in the 2nd part of the Fig. 3 for the novel SO-PSDDM. The overlapping of the results (worst, best and mean) is performed for each problem of the novel SO-PSDDM. These accurate calculations label the brilliance of the proposed computational stochastic approach. The comparison plots are drawn in Fig. 3 (g) based on the AE that are calculated as 10^–05^-10^–07^, 10^–05^-10^–06^ and 10^–04^-10^–06^ for 1st, 2nd and 3rd problem. The statistical operator performances based on the MSC, TIC and Fitness are provided in Fig. 3(h) for the novel SO-PSDDM. The best achieved values of the fitness are performed as 10^–9^-10^–10^, 10^–09^-10^–10^, 10^–08^-10^–09^ for problem 1, 2, and 3 of the novel SO-PSDDM. The operators MSE and TIC performances lie as 10^–9^-10^–10^ for problem 1 and 2, while 10^–09^-10^–10^ for problem 3 of the novel SO-PSDDM of the novel SO-PSDDM. These precise and accurate measures designated the correctness of the proposed solver for the novel SO-PSDDM.

**Figure 4:**
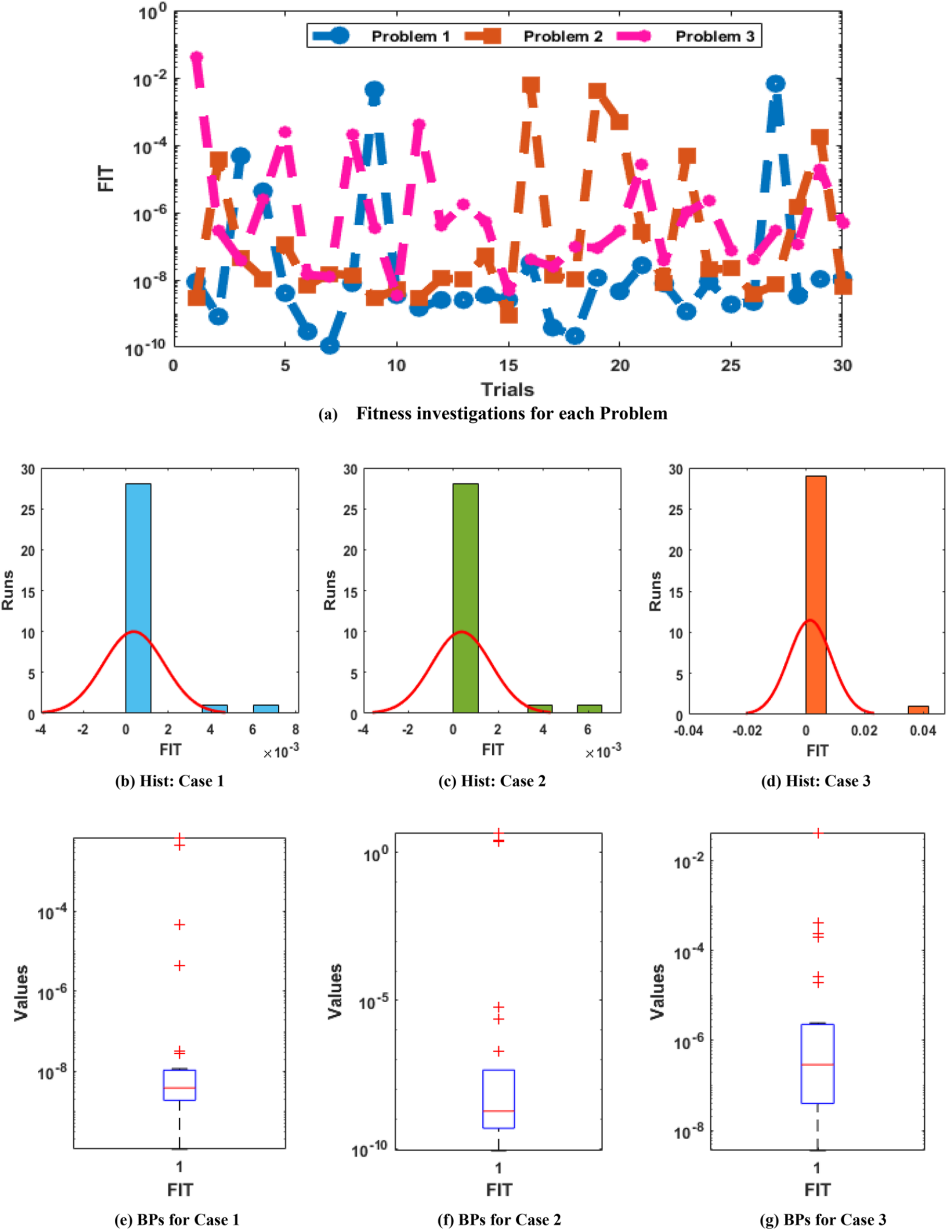
Statistical illustrations via swarming schemes based on the fitness for solving the novel SO-PSDDM.

**Figure 5:**
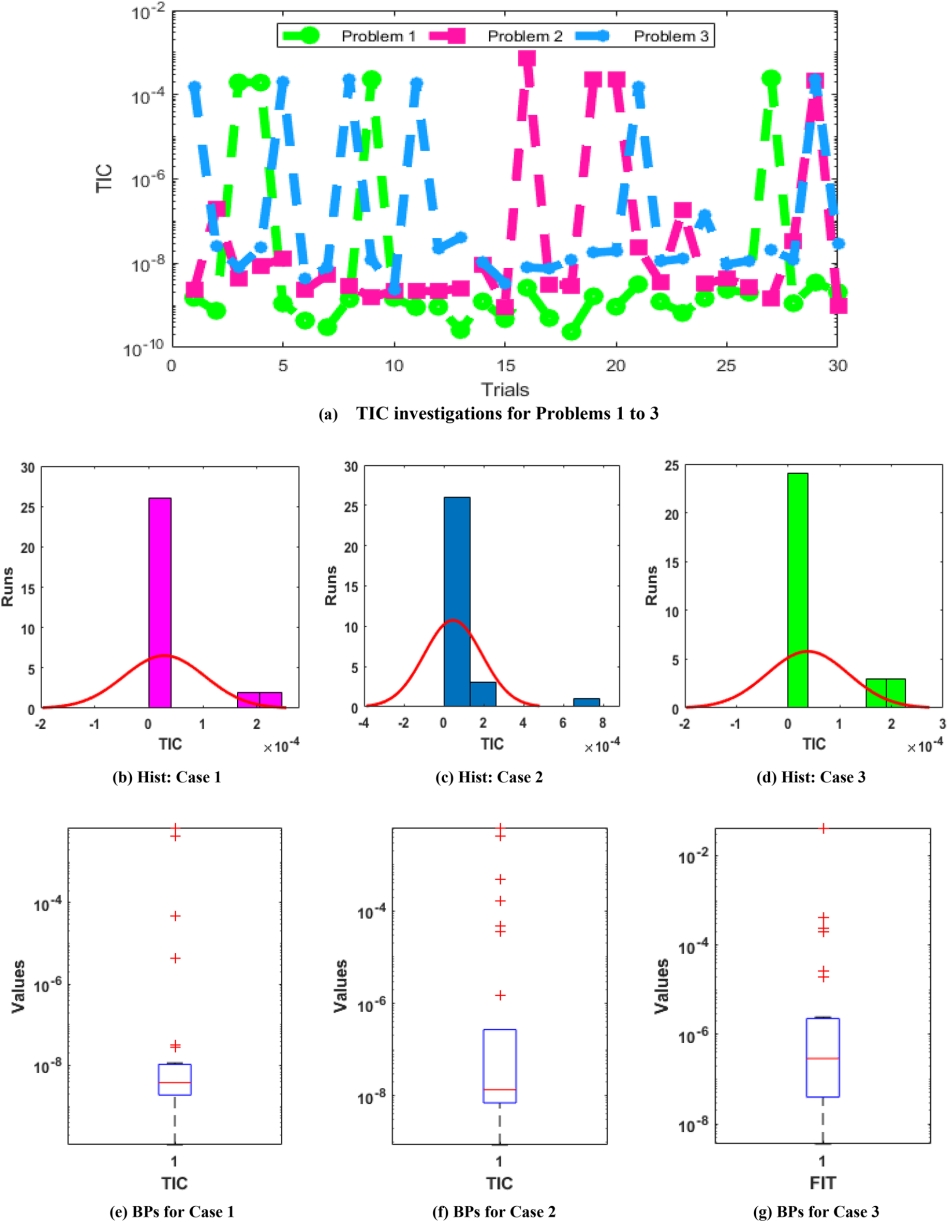
Statistical illustrations via swarming schemes based on the TIC for solving the novel SO-PSDDM.

**Figure 6:**
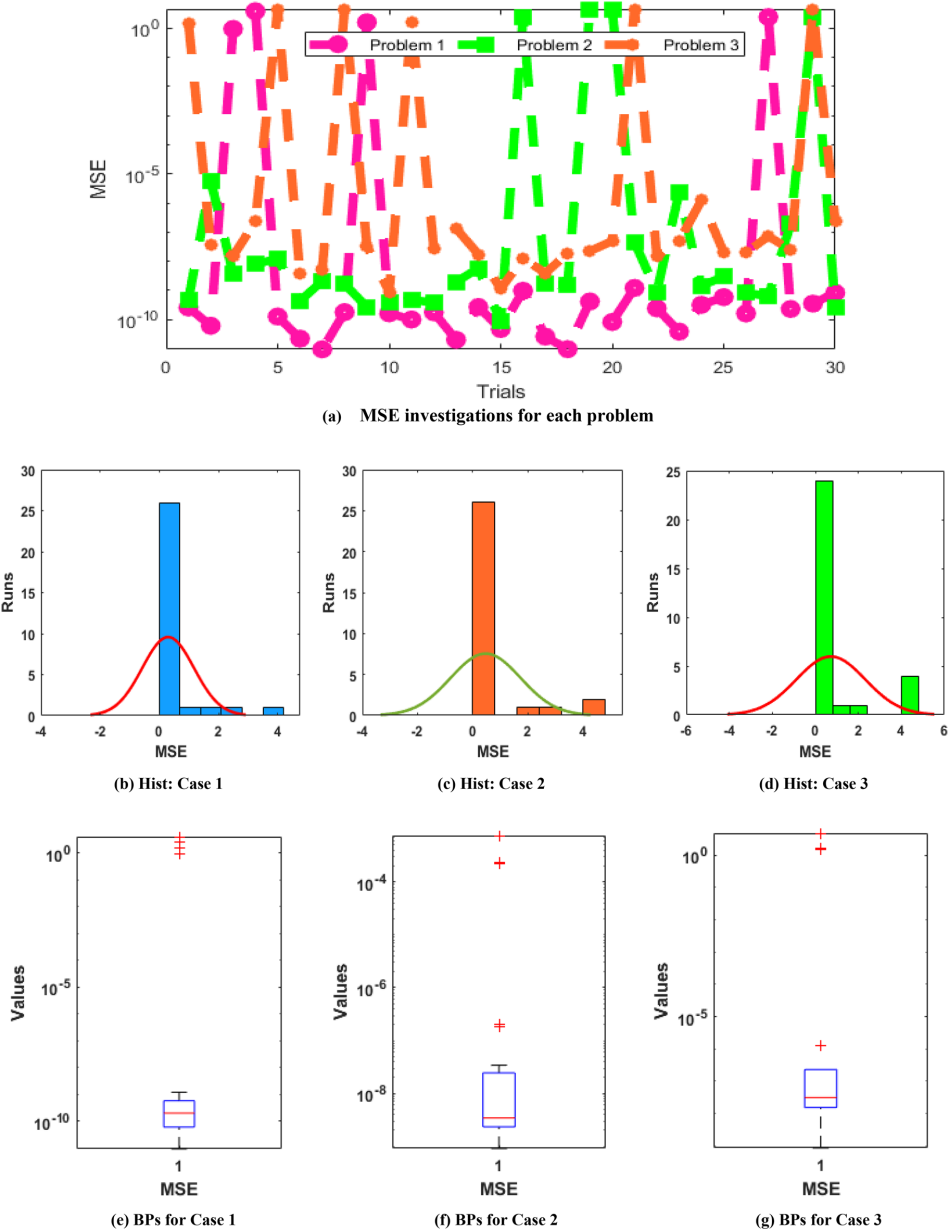
Statistical illustrations via swarming schemes based on the MSE for solving the novel SO-PSDDM.

The illustrations based on the convergence are provided in Figs. 4, 5, 6 based on TIC operator, Fitness and MSE measures using the histogram (Hist) and boxplots (BPs). The best performances of the fitness are observed in Fig. 4, which are presented as 10^–06^ to 10^–10^, 10^–06^ to 10^–08^ and 10^–06^ to 10^–07^ for problem 1, 2, and 3 of the novel SO-PSDDM. The TIC measures are performed in Fig. 5 that are obtained as 10^–08^-10^–10^, 10^–07^-10^–09^ and 10^–06^-10^–09^ for problem 1 to 3 of novel SO-PSDDM. Similarly, the MSE values are shown in Fig. 6 that are achieved as 10^–10^ to 10^–11^, 10^–07^ to 10^–10^ and 10^–06^ to 10^–10^ for problem 1, 2, and 3 of the novel SO-PSDDM. These best obtained values via statistical gages authenticate the reliability of the stochastic swarming computational schemes.

To authenticate the convergence and accuracy of the proposed stochastic swarming computational scheme, the statistical values have been performed based on the minimum (Min), Median, SIR, Mean, Standard deviation (STD) gages. These measures have been performed Tables 1, 2, 3 for thirty independent executions to solve the novel SO-PSDDM. The accurate performances of these operators’ label the accuracy and constancy of the proposed stochastic swarming computational scheme for solving the novel SO-PSDDM.

**Table 1:** Statistical presentations via stochastic performances for solving the novel SO-PSDDM-based Problem 1.

*u*	Min	Med	Mean	SIR	STD
0	3.25928E−07	4.66616E−06	9.53434E−04	2.41607E−06	4.93283E−03
0.05	6.67252E−08	4.77692E−06	1.13927E−03	4.81387E−06	5.32610E−03
0.1	2.07402E−07	5.46945E−06	1.67515E−03	6.10533E−06	6.23077E−03
0.15	4.05190E−08	6.12370E−06	2.45481E−03	6.79540E−06	7.59667E−03
0.2	5.99439E−07	6.84725E−06	3.04849E−03	6.20632E−06	8.60692E−03
0.25	5.47989E−07	9.74012E−06	2.62003E−03	5.54247E−06	7.89073E−03
0.3	1.18973E−07	9.41275E−06	3.13279E−03	7.95664E−06	1.01843E−02
0.35	8.52329E−08	7.86144E−06	8.36886E−03	7.11166E−06	2.39926E−02
0.4	3.09995E−07	6.28085E−06	1.26811E−02	7.80690E−06	3.89698E−02
0.45	6.74997E−07	5.56770E−06	9.04074E−03	6.27895E−06	2.97933E−02
0.5	8.06805E−08	3.05818E−06	1.25960E−02	4.90873E−06	4.20323E−02
0.55	3.98308E−08	3.12062E−06	4.89098E−02	3.11601E−06	1.60262E−02
0.6	1.54915E−07	2.89889E−06	8.91168E−02	5.55410E−06	3.03817E−02
0.65	3.12993E−07	6.20114E−06	1.39926E−02	6.90784E−06	4.98168E−02
0.7	1.14665E−07	7.85856E−06	2.29761E−02	7.32446E−06	7.91550E−02
0.75	1.88195E−07	6.93564E−06	3.78500E−02	4.60432E−06	1.19116E−02
0.8	3.07894E−07	1.23612E−05	5.25783E−02	1.29801E−05	1.53449E−02
0.85	4.45512E−06	2.75504E−05	5.89336E−02	1.99353E−05	1.59780E−02
0.9	3.09392E−06	3.45237E−05	5.51525E−02	2.55173E−05	1.57473E−02
0.95	1.16738E−06	1.68189E−05	6.52558E−02	2.09277E−05	1.77453E−02
1	7.08528E−06	7.36480E−05	7.95988E−02	5.47937E−05	2.07721E−02

**Table 2:** Statistical presentations via stochastic performances for solving the novel SO-PSDDM-based Problem 2.

*u*	Min	Med	Mean	SIR	STD
0	3.91605E−07	9.26747E−06	5.07470E−04	2.24202E−05	1.93356E−03
0.05	6.29302E−07	1.28941E−05	6.83530E−03	4.21742E−05	1.89956E−03
0.1	7.63730E−07	2.41048E−05	2.84035E−02	4.23485E−05	8.00115E−03
0.15	2.13225E−07	3.86512E−05	6.57355E−02	5.08616E−05	1.88075E−03
0.2	9.42074E−07	6.24658E−05	1.14787E−01	9.13037E−05	3.35634E−03
0.25	2.73184E−06	7.48152E−05	1.68132E−01	1.57460E−04	5.05306E−02
0.3	4.50193E−06	6.09892E−05	2.22950E−01	2.14585E−04	6.72256E−03
0.35	3.05625E−06	5.58300E−05	2.73928E−01	2.41463E−04	8.11942E−03
0.4	4.78660E−07	3.73123E−05	3.06411E−01	1.42908E−04	9.08282E−03
0.45	6.54305E−07	2.63053E−05	3.14441E−01	1.66944E−04	9.53662E−03
0.5	8.35949E−07	3.23571E−05	3.23737E−01	1.63346E−04	9.44918E−03
0.55	5.81528E−08	2.87395E−05	3.28815E−01	4.28922E−05	9.03771E−03
0.6	3.76732E−06	2.08270E−05	3.27214E−01	3.12623E−05	8.58655E−03
0.65	8.54568E−07	1.59875E−05	3.20494E−01	5.10999E−05	8.32470E−03
0.7	3.26665E−06	3.75830E−05	3.11436E−01	4.25909E−05	8.32622E−02
0.75	4.86321E−06	5.21953E−05	3.05148E−01	4.75391E−05	8.52543E−02
0.8	3.02292E−06	4.94802E−05	3.08327E−01	2.94906E−05	8.86728E−02
0.85	2.60173E−06	3.57292E−05	3.24533E−01	1.01432E−04	9.40117E−02
0.9	2.76980E−06	8.71741E−05	3.48362E−01	9.62001E−05	1.01498E−02
0.95	1.48408E−06	1.29774E−04	3.74016E−01	1.35267E−04	1.10562E−02
1	6.47615E−07	5.20566E−05	4.03910E−01	1.59708E−04	1.21609E−02

**Table 3:** Statistical presentations via stochastic performances for solving the novel SO-PSDDM-based Problem 3.

*u*	Min	Med	Mean	SIR	STD
0	2.81494E−07	5.38021E−05	4.12286E−04	3.45392E−05	1.81281E−03
0.05	3.97733E−07	5.45424E−05	2.19502E−02	1.14050E−04	4.96580E−02
0.1	3.58538E−06	1.63741E−04	8.15904E−02	2.02824E−04	1.80321E−01
0.15	4.02099E−05	2.95543E−04	1.60298E−01	3.47039E−04	3.51184E−01
0.2	4.31225E−06	3.92231E−04	2.34821E−01	6.60673E−04	5.24591E−01
0.25	5.76076E−05	4.67731E−04	2.84154E−01	6.21411E−04	6.77294E−01
0.3	1.13333E−05	4.66191E−04	3.34891E−01	2.40195E−04	7.76930E−01
0.35	7.41801E−07	3.35635E−04	3.46584E−01	2.38453E−04	8.30033E−01
0.4	1.66043E−05	2.32169E−04	3.39964E−01	1.63506E−04	8.35122E−01
0.45	1.24690E−05	1.71361E−04	3.31192E−01	2.12338E−04	8.09979E−02
0.5	1.20792E−05	1.02758E−04	3.22086E−01	2.88302E−04	7.72343E−02
0.55	6.84075E−06	1.40519E−04	3.12250E−01	2.44493E−04	7.40156E−02
0.6	5.97871E−06	1.39992E−04	3.25147E−01	2.43317E−04	7.49184E−02
0.65	2.66925E−05	1.28135E−04	3.86269E−01	1.04294E−04	8.16101E−02
0.7	9.74861E−06	9.33194E−05	4.52305E−01	1.34891E−04	9.27394E−02
0.75	1.54588E−06	1.29861E−04	5.16051E−01	2.19343E−04	1.05123E−01
0.8	3.24905E−06	1.55802E−04	5.76658E−01	2.17065E−04	1.17445E−01
0.85	1.39944E−05	1.23407E−04	6.37357E−01	9.91446E−05	1.29858E−01
0.9	3.80092E−06	1.05567E−04	6.98774E−01	1.71256E−04	1.42311E−01
0.95	1.74201E−05	2.13083E−04	7.37263E−01	2.81903E−04	1.50103E−01
1	2.83864E−06	4.32771E−05	6.87038E−01	1.04323E−04	1.54045E−01

The convergence illustrations via the proposed stochastic swarming computational scheme using the global form of the fitness, TIC and MSE for 30 trials are illustrated in Table 4 to solve the novel SO-PSDDM. The Min performances of the Global Fitness, TIC are reported as 10^–03^-10^–04^, 10^–03^ to 10^–04^, while for MSE these performances are 10^–04^ to 10^–06^. Whereas the SIR for these gages found as 10^–06^ to 10^–09^, 10^–03^ to 10^–04^ and 10^–08^ to 10^–10^. The best global performances validate the exactness of stochastic scheme for the SO-PSDDM.

**Table 4:** Global representations by using the stochastic performances for solving the novel SO-PSDDM-based Problem 3.

Index	Problem	G.FIT		G. TIC		G.MSE	
		MIN	SIR	MIN	SIR	MIN	SIR
$\hat{z}(u)$	1	3.7152E−04	4.3797E−09	1.9329E−03	8.1786E−06	2.8839E−05	7.9659E−10
	2	3.6592E−04	1.3136E−07	2.4658E−04	9.5087E−05	4.6636E−05	1.1046E−08
	3	1.3656E−03	1.1248E−06	3.7081E−03	1.7854E−04	3.8046E−05	1.5468E−08

The complexity cost performances for each case of the novel SO-PSDDM using the stochastic computing performances based on the iterations, executed time along with the function measures is provided in Table 5. The average iterations, implementation of time along with the count of function are calculated as 39.53151, 996.64444 and 88,684.355555, respectively for the novel SO-PSDDM using the stochastic procedures.

**Table 5:** Complexity performances for each case of the for solving the novel SO-PSDDM.

Problem	Iterations		Executed time		Function computations	
	Mean	STD	Mean	STD	Mean	STD
1	44.4870978	10.13863940	1005.0000	138.3050244	99,753.53333	21,816.36993
2	35.18988877	10.29969406	979.93333	137.2957877	78,988.40000	23,091.78820
3	38.91754381	10.69665569	1005.0000	136.9856497	87,311.13333	23,301.85795

## Concluding remarks

In this work, a mathematical perturbed delay differential singular model is designed by using the standard Lane-Emden model. The inclusive structures based on the delay terms, singular-point and perturbation factor have been provided along with the shape factor based on the SO-PSDD system. The novel system represents the singularity at one point, whereas the delay and perturbed factors have been noted twice. The singular form of the system becomes more complicated with the perturbed/delay terms. The novel SO-PSDDM is numerically simulated by using the artificial neural networks along with the optimization measures of the swarming procedures and interior-point algorithm. An error function is optimized through the swarming PSO procedure and IPA to solve the SO-PSDDM. The precision, substantiation and validation have been observed for three problems. The correctness of the novel SO-PSDDM has been observed by comparing the obtained and exact solutions. For the reliability, stability and convergence of the proposed scheme, the statistical performances of the Min, Median, SIR, Mean, Standard deviation STD gages have been observed for 30 independent trials. Additionally, the new model become complicated with the delay, perturbed and singular terms. Therefore, these systems are not easy to solve by using the traditional scheme. Therefore, the artificial intelligence based swarming scheme is a suitable procedure to deal such complex and harder nature systems.

In upcoming investigations, the novel designed perturbed delay differential model will be solved by using the Morlet wavelet, Meyer wavelet and Gudermannian neural networks^63–68^.

## Data Availability

The datasets generated/produced during and/or analyzed during the current study/research are available from the corresponding author on reasonable request.
